# Deep Brain Stimulation in Moroccan Patients With Parkinson's Disease: The Experience of Neurology Department of Rabat

**DOI:** 10.3389/fneur.2018.00532

**Published:** 2018-07-31

**Authors:** Mounia Rahmani, Maria Benabdeljlil, Fouad Bellakhdar, Mustapha El Alaoui Faris, Mohamed Jiddane, Khalil El Bayad, Fatima Boutbib, Rachid Razine, Rachid Gana, Moulay R. El Hassani, Nizar El Fatemi, Meryem Fikri, Siham Sanhaji, Hennou Tassine, Imane El Alaoui Balrhiti, Souad El Hadri, Najwa Ech-Cherif Kettani, Najia El Abbadi, Mourad Amor, Abdelmjid Moussaoui, Afifa Semlali, Saadia Aidi, El Hachmia Ait Benhaddou, Ali Benomar, Ahmed Bouhouche, Mohamed Yahyaoui, Abdeslam El Khamlichi, Abdessamad El Ouahabi, Rachid El Maaqili, Houyam Tibar, Yasser Arkha, Adyl Melhaoui, Abdelhamid Benazzouz, Wafa Regragui

**Affiliations:** ^1^Research Team in Neurology and Neurogenetics, Department of Neurology A and Neuropsychology, Faculty of Medicine and Pharmacy, Hôpital des Spécialités ONO, University Mohammed V, Rabat, Morocco; ^2^Department of Neurosurgery, Faculty of Medicine and Pharmacy, Hôpital Ibn Sina, University Mohammed V, Rabat, Morocco; ^3^Department of Neuroradiology, Faculty of Medicine and Pharmacy, Hôpital des Spécialités ONO, University Mohammed V, Rabat, Morocco; ^4^Research Team in Neurology and Neurogenetics, Department of Neurology B and Neurogenetics, Faculty of Medicine and Pharmacy, Hôpital des Spécialités ONO, University Mohammed V, Rabat, Morocco; ^5^Laboratory of Biostatistics, Clinical Research and Epidemiology, Faculty of Medicine and Pharmacy, University Mohammed V, Rabat, Morocco; ^6^Department of Anesthesia and Intensive Care, Faculty of Medicine and Pharmacy, Hôpital des Spécialités ONO, University Mohammed V, Rabat, Morocco; ^7^Department of Surgical Intensive Care, Faculty of Medicine and Pharmacy, Hôpital Ibn Sina, University Mohammed V, Rabat, Morocco; ^8^Department of Neurosurgery, Faculty of Medicine and Pharmacy, Centre de Rehabilitation et de Neurosciences, Hôpital des Spécialités ONO, University Mohammed V, Rabat, Morocco; ^9^Centre National de la Recherche Scientifique, Institut des Maladies Neurodégénératives, Univ. de Bordeaux UMR 5293, Bordeaux, France

**Keywords:** Parkinson disease, deep brain stimulation, subthalamic nucleus, quality of life, surgical benefit, clinical outcome

## Abstract

**Introduction:** Deep brain stimulation (DBS) of the subthalamic nucleus (STN) is known as a therapy of choice of advanced Parkinson's disease. The present study aimed to assess the beneficial and side effects of STN DBS in Moroccan Parkinsonian patients.

**Material and Methods:** Thirty five patients underwent bilateral STN DBS from 2008 to 2016 in the Rabat University Hospital. Patients were assessed preoperatively and followed up for 6 to 12 months using the Unified Parkinson's Disease Rating Scale in four conditions (stimulation OFF and ON and medication OFF and ON), the levodopa-equivalent daily dose (LEDD), dyskinesia and fluctuation scores and PDQ39 scale for quality of life (QOL). Postoperative side effects were also recorded.

**Results:** The mean age at disease onset was 42.31 ± 7.29 years [28–58] and the mean age at surgery was 54.66 ± 8.51 years [34–70]. The median disease duration was 11.95 ± 4.28 years [5–22]. Sixty-three percentage of patients were male. 11.4% of patients were tremor dominant while 45.71 showed akinetic-rigid form and 42.90 were classified as mixed phenotype. The LEDD before surgery was 1200 mg/day [800-1500]. All patients had motor fluctuations whereas non-motor fluctuations were present in 61.80% of cases. STN DBS decreased the LEDD by 51.72%, as the mean LEDD post-surgery was 450 [188-800]. The UPDRS-III was improved by 52.27%, dyskinesia score by 66.70% and motor fluctuations by 50%, whereas QOL improved by 27.12%. Post-operative side effects were hypophonia (2 cases), infection (3 cases), and pneumocephalus (2 cases).

**Conclusion:** Our results showed that STN DBS is an effective treatment in Moroccan Parkinsonian patients leading to a major improvement of the most disabling symptoms (dyskinesia, motor fluctuation) and a better QOL.

## Introduction

Stereotactic surgery represents a highly effective therapy for the treatment of Parkinson's disease (PD) and other movement disorders refractory to medical treatment. The use of deep brain stimulation (DBS) for PD was driven by advances in the understanding of the pathophysiology and availability of animal models of the disease. In 1993, Benazzouz et al. (1) successfully performed high frequency stimulation of the subthalamic nucleus (STN) in Macaca mulatta monkeys rendered parkinsonian by MPTP (1-methyl-4phenyl-1,2,3,6-tetrahydropyridine).The authors have reported dramatic improvements of the motor symptoms without the development of abnormal involuntary movements. In 1994, Benabid et al. (2) and Siegfried and Lippitz (3) reported successful treatment of patients with PD who underwent DBS of the subthalamic nucleus (STN) and of the globus pallidus internus (GPi), respectively. The procedure is now commonly used in patients with intractable tremor, or with disabling drug-induced complications, especially motor fluctuations and/or dyskinesia. DBS in its current form is a symptomatic treatment that does not interfere with the progression of the disease, and does not affect the non-levodopa responsive motor and non-motor aspects of the disorder such as levodopa-refractory freezing of gait and balance problems nor non-motor aspects of the disease (4).

Nowadays, the STN is widely considered the target of choice (5–8). The mechanism of the stimulation effect on PD is not fully understood but thought to likely be related to the modulation of neuronal activity and the reinstatement of balance within basal ganglia connections (4, 9, 10). The postoperative clinical outcome depends on the quality of the inclusion clinical criteria and the precision of targeting for electrode implantation, which is based on neuroimaging techniques, intraoperative electrophysiology and test of the stimulation effects (11, 12).

Multiple series have reported on the long-term efficacy of DBS for PD (13–23). Here we report our experience of STN DBS performed in a cohort of Moroccan PD patients over a period of 9 years. We describe our results of this first Moroccan series, with a particular emphasis on evaluating the effectiveness and safety of this neurosurgical treatment on the first year of follow up.

## Materials and methods

We conducted a retrospective study of 35 patients with advanced PD who underwent bilateral STN DBS surgery from January 2008 to December 2016 in the University Hospital of Rabat. Surgery was performed in two different departments of neurosurgery, using the same technical procedure. The study was approved by ethics committee of medical school of Rabat and all patients provided their written informed consent.

### Patient's selection

Evaluations were performed by neurologists specialized in movement disorders. Inclusion criteria for pre-operative assessment were diagnosis of PD according to the UK Brain Bank Criteria (24), age under 70 years old, severe parkinsonian motor symptoms or dyskinesia that limit activities of daily living despite optimal medical therapy for at least 6 months, no dementia or major general illness. A systematic psychiatric appraisal was made to assess and treat any severe depression.

All patients underwent a pre-operative testing. Levodopa challenge was analyzed, and an improvement of at least 30% was necessary to confirm levodopa responsiveness. A morphologic MRI was performed to exclude patients with severe cerebral atrophy, ischemic lesions or other brain injuries that may contra-indicate the surgical procedure. All subjects had also a neuropsychological testing. Global cognitive functioning was evaluated by the Mattis Dementia Rating Scales (MDRS) (25, 26) and the Montreal Cognitive Assessment (MoCA) (26, 27), intellectual capacities by the Progressive Matrices de Raven (PM47) (26, 28), executive functions by the Trail Making Test (TMT), Stroop test and Frontal Assessment Battery (FAB), memory by the verbal fluency and the Memory Impairment Screen (MIS-D), visual and constructive abilities by the Rey figure and the Benton Visual Retention Test (26). We considered a score of MDRS lower than 130/144 a cut off for DBS eligibility as well as severe executive troubles. Patients were eligible for surgery if they responded to levodopa, challenge with normal brain MRI, normal cognitive tests, and no major drug-resistant depression.

#### Pre-operative evaluation

All patients were assessed using a form that specifies the demographic characteristics (age, gender, age of onset, duration of the disease), clinical features [laterality of symptoms, predominant features: tremulous, akinetic-rigid, or mixed subtypes according to criteria used in 2008 by Rajput et al. (29)], disease severity assessed by the motor section of the UPDRS score (Unified Parkinson's Disease Rating Scale) and Hoehn and Yahr scale (24), motor complications [fluctuations, dyskinesia, freezing of gait (FOG)] and non-motor fluctuations (dysautonomic troubles, sleep disorders, depression, cognitive disturbances, hallucinations, delirium), Levodopa Equivalent Daily Doses (LEDD) calculated based on a previously published algorithm combining dopamine agonist daily dose with levodopa daily dose (30), UPDRS I for mental cognitive assessment, UPDRS II and Schwab and England Scale for activity of daily living (ADL), Giovannoni criteria (31) for dopamine dysregulation syndrome (DDS), Montgomery-Asberg Depression Scale (MADRS) and Hamilton anxiety rating scale (HAM-A) to evaluate mood disorder and PDQ 39 for quality of life (32).

### Surgical targeting and procedure

A Leksell G stereotactic frame (Elekta AB) was placed under local anesthesia. We performed a preoperative 1.5 Tesla cerebral MRI with the following sequences: ventriculographic CISS (constructive interference in steady state), MPR (Multi-plan Reconstruction) with gadolinium, and coronal T2 DESS (double echo steady state) followed by a CT scan to check for any MRI-generated distortion. All the images were transferred to the surgical planning station (Elekta Surgiplan^*^). The STN coordinates were calculated using direct (based on MRI T2 DESS) and indirect (using statistical coordinates) methods (33, 34).

The first operated side was the one contralateral to the most impaired body-side. The electrodes were implanted in a single operative session under local anesthesia and the target was identified by a combination of neuroimaging, microelectrode recording, and stimulation tests. For each patient, trajectories were determined on the basis of individual anatomical variations and stereotactic MRI based software was used to plan an optimal trajectory from the defined entry point to the sensorimotor STN stimulation target (anatomically referred to as dorso-lateral STN) by avoiding critical brain structures.

The STN stimulation target was defined using a combination of statistical coordinates of STN (4 mm inferior, 3.9 posterior, and 12 mm lateral from midcomissural point), and direct visualization on MRI where the STN was chosen at the anterior margin of red nucleus and 2 to 3 mm lateral from its external border.

Stereotactic gadolinium enhanced T1-weighted images were used to visualize vessels to avoid injury of any vascular structure during surgery.

All trajectories were anterior to the motor strip close to the coronal suture on sagittal plane, and about 2 to 3 cm from midline in coronal plane. The final trajectory was defined as to get a maximum of definitive electrode plots within the visualized hypointensity of STN. To reach this, the trajectory direction should superimpose to the vertical axis of the STN.

Multi-track (3–5) microelectrodes were inserted for electrophysiological mapping of the STN. Subsequent macro-stimulations were used to assess the efficacy and side effect profile of the tested electrodes. The optimal track (best micro-recording and widen therapeutic window on macro-stimulation) was chosen for each side and the permanent quadripolar leads were implanted (model 3389; Medtronic, Minneapolis, Minn., USA). Continuous fluoroscopy was used to monitor a potential electrode displacement and to confirm the definitive electrode positioning. Non-absorbable silk stich was used to anchor the definitive electrode and the burr hole was sealed by acrylic cement. The mean surgery duration was 5–7 h from the scalp incision.

Post-operative CT scan was performed immediately after surgery to rule out surgical complications such as hemorrhage and to confirm the final location of the implanted electrodes based on fusion of the preoperative MRI and postoperative CT scan images. The internal pulse generators (model 7428 Kinetra, or 37601 Activa PC; Medtronic) were implanted the same day or 3–5 days later in a subcutaneous pocket in the infra-clavicular region under general anesthesia.

### Stimulation programming

The 8 lead contacts were assessed 1–3 months after surgery when the lesion-like effect responsible of spontaneous postoperative improvement of PD symptoms, has disappeared. The best contact that improved the symptoms without side effects was chosen for each side.

### Post-operative evaluation

Patients were evaluated 6–12 months after surgery using section III of the UPDRS in four conditions (stimulation OFF and ON and medication OFF and ON). Subscales of UPDRS III were assessed as follow: speech score expresses item 18, tremor is the sum of items 20, and 21, rigidity is item 22, bradykinesia is the sum of items 23 to 27, 30, and 31, posture is item 28 and postural instability item 29. Patients were also assessed for the LEDD, dyskinesia score (the sum of items 32–35 of the UPDRS IV), motor fluctuations score (the sum of items 36-39 of the UPDRS IV), MADRS, HAM-A and PDQ39 scale expressed as summary index (SI) that ranges between 0 and 100% (100% is equivalent to bad quality of life) and as its eight dimensions (35). Postoperative side effects were also recorded.

### Statistical analysis

SPSS 13.0 software was used for the statistical processing of our data. Quantitative data were expressed in mean ± standard deviation (*SD*) or median and interquartile range. Categorical variables were expressed as numbers and percentages. Data were tested for normal distribution by graphical methods. Pre and post intervention quantitative variables of normal distribution were compared using paired-t test (UPDRS III, Dyskinesia scores, Motor Fluctuations scores, PDQ39 SI, Bradykinesia subscale of UPDRS III) and pre and post intervention quantitative variables of non normal distribution (UPDRS I, UPDRS II, LEDD, PDQ 39 subscales, speech, tremor, rigidity, posture, and postural instability subscales of UPDRS III) and ordinal variables (UPDRS V and VI) were analyzed using Wilcoxon test. Pre and post intervention categorical data were compared using McNemar test. Statistical significance was assumed for tests yielding *p*-values of less than 0.05.

## Results

### Demographic data

The mean age at disease onset was 42.31 ± 7.29 years and the mean age at surgery was 54.66 ± 8.51 years. The median disease duration was 11.95 ± 4.28 years. Sixty-three percentage of patients were male. Four patients were tremor-dominant, 16 patients showed an akinetic rigid form and 15 patients were classified as mixed subtype. The LEDD before surgery was 1200 mg/day [800-1500]. Mean time to appearance of motor fluctuations was 7.81 ± 4.25 years [1–16], non-motor fluctuations 8.05 ± 3.34 years [2–15] and dyskinesia 7.82 ± 3.76 years [2–14]. The most disabling symptom was akinetic state (40%) followed by akinesia and dyskinesia in the same time (34.21%) then dyskinesia alone (7%). Only one patient suffered from disabling tremor associated with dyskinesia. More than one third of patients presented a dopamine dysregulation syndrome (Table 1).

**Table 1:** Baseline characteristics of the study population (*n* = 35).

**Clinical data**	**Figures**
Age at onset	42.31 ± 7.29 [28–58]^**^
Age at surgery	54.65 ± 8.51 [34–70]^**^
Sex (M)	62.90 (22)^*^
Disease duration	11.97 ± 4.28 [5–22]^**^
Motor phenotype	
Akinetic-rigid	45.70 (16)^*^
Tremoric	11.40 (4)^*^
Mixed	42.90 (15)^*^
Motor complications	
Motor fluctuations	100 (35)^*^
Dyskinesia	85.71(30)^*^
Freezing of gait	28.57 (10)^*^
Non-motor fluctuations	61.80 (21)^*^
Most disabling symptoms	
Akinesia	40.00 (14)^*^
Dyskinesia	20.00 (7)^*^
Akinesia and dyskinesia	34.28 (12)^*^
Tremor and dyskinesia	2.85 (1)^*^
DDS	34.28 (12)^*^
LEDD	1200 [800–1500]^***^

* *Percentage (number)*,

** *Means and standard deviation [minimum, maximum]*,

*** *Median and interquartile range, M, males; DDS, dopamine dysregulation syndrome; LEDD, levodopa equivalent daily doses*.

### Surgery related complications

Some side effects were recorded during or immediately after surgery. They were mild and transient: confusional episode (2 cases), hallucination (1 case), hypophonia (1 case), aphonia (1 case), anxiety (2 cases), hypomania (1 case), and pneumocephalus (2 cases).

**DBS Related Complications**

Long term complications were as follow: two patients suffered from dysarthria that needed changes of stimulation parameters, three patients presented direct hardware-related complications; two patients exhibited a battery site infection 3 and 5 months after surgery that was resolved after antibiotic treatment for one patient and after removal of the battery and the lead for the other one. Another subject experienced, 6 months after surgery, an infection of the lead/wire connection site. The whole DBS system was removed then repositioned 8 months later. One patient had unilateral misplacement of the lead with a medial deviation of 3.2 mm. Following a reimplantation of the lead, the patient presented clinical improvement and the CT scan showed correct positioning of the lead.

### Changes in motor outcome

Comparison between pre-and postoperative clinical state is summarized in Table 2. There is no change in the part II of the UPDRS following surgery except for a tendency to a small improvement in activity of daily living during OFF medication state up to 10% but without reaching significance (*p* = 0.06).

**Table 2:** Comparison of pre and post-operative clinical state (stimulation ON).

**Clinical data**	**Preoperative**	**Postoperative**	**% of improvement**	***P-*value**
UPDRS I (range 0–16)	3 (1–4) *2.80 ± 1.80*	2 (1–4) *2.35 ± 1.67*	37.50 [−27.08 to 68.75] *3.63 ± 100.48*	0.190
**MEDICATION OFF**				
UPDRS II (0-52)	23 [13.50–31.50] *21.88 ± 9.64*	17 [11.00–20.25] *16.80 ± 7.05*	10.00 [−9.79 to 64.86] −*8.11 ± 105.51*	0.068
UPDRS III (0–108)	45 (37–58) *45.97 ± 14.21*	20 (12–29) *22.2 ± 11.3*	52.27 [35.29–65.21] *47.50 ± 30.12*	<**0.001**
UPDRS V (0–5)	3 (3, 4) *3.38 ± 0.87*	3 [2.5–3] *3.07 ± 0.90*	0 [0–16.67] *1.98 ± 29.30*	0.323
UPDRS VI (0–100%)	40% [20–60%] *39.00 ± 21.87%*	70% [37.5–80%] *60.81 ± 24.71%*	36.66 [0.00–150.00] *104.71 ± 201.88*	**0.013**
FOG	20.60(7)	0(0)		**0.016**
**MEDICATION ON**				
UPDRS II (0–52)	6.00 [2.50–14.00] *9.24 ± 8.98*	6.50 [3.75–10.00] *7.54 ± 5.56*	0[−55.47 to 69.64] −*55.47 ± 189.93*	0.808
UPDRS III (0–108)	10 (7–13) *10.71 ± 5.22*	8 [5.5–18.5] *11.5 ± 8.6*	14.28[−87.50 to 58.11] −*33.50 ± 120.84*	0.788
UPDRS V (0–5)	2 [1.5–2.5] *1.90 ± 0.86*	2 [1.5–2.5] 2 ± 0.56	0 [0–25] −*2.8 ± 44.89*	0.898
UPDRS VI (0–100%)	80% [70–90%] *78.24 ± 23.78%*	85% [80–100%] *83.12 ± 20.58%*	5.56 [−10.83 to 22.32] *7.67 ± 23.11*	0.152
Dyskinesia score (range 0–1)	6 [3.25–7.75] *5.75 ± 2.57*	2 (1–3) *2.25 ± 1.77*	66.70 [32.50–74.10] *58.70 ± 27.84*	<**0.001**
MF Score (range 0–7)	4 (4, 5) *4.2 ± 1.1*	2 (2, 3) *2.1 ± 1.1*	50[6.2–60] 44.9 ±33.5	**0.001**
LEDD (mg/j)	1200 [800–1500] *1237.60 ± 577.37*	450 [188–800] *511.87 ± 368.11*	51.72 [34.28–80.77] *54.36 ± 30.02*	<**0.001**
PDQ−39 SI (0–100%)	40.00 [30.00–47.00] *38.03 ± 12.88*	27.40 [17.80–34.1] *28.62 ± 11.91*	27.12 [8.61–38.21] *10.35 ± 66.28*	**0.003**
MADRS				
<7	32(8)	44(11)		0.549
>7	68(13)	56(14)		
HAM-A	40 (10)	60 (15)		1.000
DDS	34.28(12)	25.71(9)		1.000
LEDD (mg/j)	1200 [800–1500] *1237.60 ± 577.37*	450 [188–800] *511.87 ± 368.11*	51.72 [34.28–80.77] *54.36 ± 30.02*	<**0.001**

*Data are expressed as means ± standard deviation or median and interquartile range [25–75%], or percentage (number), MF, motor fluctuation; FOG, freezing of gate; LEDD, levodopa equivalent daily doses; DDS, dopamine dysregulation syndrome; PDQ 39SI, PDQuestionnaire-39 summary index; MADRS, Montgomery and Asberg Depression Rating Scale; HAM-A, Hamilton anxiety scale. Bold values refer to significant p value*.

There is a significant improvement of the UPDRS III especially when patients were in OFF state with a rate of 52.25%. Motor fluctuation scores were improved by 50% and dyskinesia score by more than 66%. There was no more OFF FOG after surgery. The England and Schwab score in OFF medication was also improved by 36.66%. LEDD was decreased up to 51.72%.

UPDRS III subscales analysis in the four conditions, medication OFF and ON with and without stimulation, showed that tremor, rigidity, and bradykinesia scores were significantly improved by STN DBS in both medication OFF and ON states. Posture was improved only in medication OFF state and there was no modification in speech and postural instability by stimulation. The whole UPDRS III score was improved by 50% or more in medication OFF and ON states (Table 3).

**Table 3:** Effects of STN DBS on UPDRS III subscales.

**Scores**	**Medication OFF**			**Medication ON**		
	**STIM OFF**	**STIM ON**	**P value**	**STIM OFF**	**STIM ON**	**P-value**
Speech (item18)	1(1, 2) *1.52 ± 1.09*	1(1, 2) *1.36 ± 1.07*	0.449	1[0–2] *1.04 ± 1.07*	1[0–1] *0.88 ± 0.80*	0.317
Tremor (items 20+21)	3[0–5] *3.64 ± 4.41*	0[0–2.5] *1.32 ± 1.70*	**0.001**	0[0–3] *1.74 ± 3.25*	0[0–1] *0.83 ± 1.66*	**0.018**
Rigidity (item 22)	9[5–11.5] *8.12 ± 4.32*	4[2–6.5] *5.00 ± 3.91*	<**0.001**	3(1–8) *4.91 ± 4.50*	1[0–3] *1.88 ± 2.27*	<**0.001**
Bradykinesia (sum of items 23–27+30+31)	24 [16.5–32.5] *23.80 ± 9.32*	11 (8–19) *13.12 ± 6.56*	<**0.001**	8 (5–21) *12.70 ± 10.95*	4.5 (3–8) *6.00 ± 5.33*	<**0.001**
Posture (item 28)	1 [0–1] *0.80 ± 0.76*	1 [0–1] *0.48 ± 0.59*	**0.005**	0 [0–0] *0.26 ± 0.54*	0 [0–0] *0.13 ± 0.34*	0.083
Postural instability (item 29)	2 (1, 2) *1.75 ± 1.03*	1 (1, 2)**1.40 ± 1.04*	0.109	2 (1, 2) *1.48 ± 1.12*	1 [0–2] *1.08 ± 1.06*	0.159
UPDRS III (range 0–108)	42.00[34.00–57.00] *44.39 ± 14.72*	20.00 [12.00–29.00] *22.19 ± 11.36*	<**0.001**	18.50 [8.25–34.75] *23.79 ± 16.93*	8.00 [5.50–18.50] *11.52 ± 8.62*	<**0.001**

*Data are expressed as median and interquartile range [25–75%] and means ± standard deviation. STIM, stimulation; Paired t-test was used to compare the pre and post intervention means of UPDRS III, Bradykinesia subscale of UPDRS III. Wilcoxon test was used for the other subscores. Bold values refer to significant p value. Bold values refer to significant p value*.

### Changes in quality of life and non-motor outcome

There was no modification in UPDRS part I, depression, anxiety scores or in the prevalence of the DDS. The quality of life (PDQ-39 SI) was improved by 27%. We also assessed the subscales of PDQ-39 score. The only improved dimensions were mobility (*p* = 0.004), ADL (*p* = 0.003), and Sigma (*p* = 0.038). The others, mainly emotional well-being (*p* = 0.166), social support (*p* = 0.806), cognition (*p* = 0.954), communication (*p* = 0.747), and bodily discomfort (*p* = 0.281) were not improved.

### Post-operative DBS setting

As shown in Table 4, contact 3 (37.35%) was chosen most often for permanent stimulation followed by contact 2 (34.94%) and contact 1 (18.07%). Contact 0 was rarely selected (9.63%). Contact 0 refers to the most ventral contact and contact 3 to the most dorsal one. The mean proportion of patients who needed bipolar stimulation was 11.42% whereas 12.85% needed two active contacts. The stimulation parameters (mean ± SD) were 2.82 ± 0.57 V, 163.29 ± 35.79 Hz and 65.67 ± 11.91 μs (2 patients had 60 μs at one side and 90 μs at the other).

**Table 4:** Electrode contact chosen for permanent stimulation (35 patients).

**Contacts**	**Right STN**	**Left STN**	**TOTAL (83)**
0	4	4	8 (9.63%)
1	6	9	15 (18.07%)
2	15	14	29 (34.94%)
3	14	17	31 (37.35%)

*Data are given as numbers (%). thirteen electrodes were employed for double monopolar contact stimulation and eight electrodes for bipolar stimulation. Contact 0 refers to the most ventral contact and contact 3 the most dorsal one. STN, subthalamic nucleus*.

## Discussion

STN-DBS efficacy on PD motor symptoms is well documented in the short and medium terms, up to 5 years [(13)–(23, 36, 37)], while a few publications with a small number of examined patients addressed the long-term efficacy of this procedure (38–41). Here, we report the one-year outcome of a cohort of 35 consecutive PD patients at advanced stage of the disease, who underwent bilateral STN-DBS.

The age at surgery of 54 years was in line with a Canadian series (38) but much lower than larger series where patients were operated between 58-61 years of age (36, 37, 42). This can be explained by the young age of onsetalready reported in our population with an age of onset < 55 years in 45% and < 45 years in 15% (43). The disease duration of 11.97 ± 4.28 years was similar to other series [15 16, 22, 23, 36–39, 42, 44]. The usefulness of DBS in early stage of the disease is currently a subject of debate. Results of the EarlyStim Trial (45) demonstrated that DBS was superior to medical therapy with respect to motor disability, activities of daily living, levodopa-induced motor complications and time with good mobility and no dyskinesia. However, the poor access of our population to DBS surgery makes the question quite obsolete, as we are in the obligation to offer this therapeutic option to really disabled patients.

When compared to baseline, STN DBS in our patients allowed a major benefit in different components of motor function as widely noted in different series (46). It improved the UPDRS III score and the cardinal symptoms both in OFF and ON medication conditions. These results attest two points: (i) the superiority of DBS in relieving these symptoms as patients had better scores in ON medication/ON stimulation than in ON medication/OFF stimulation and (ii) the best motor state in the morning before taking their first dose of dopaminergic drugs (OFF medication/ON stimulation). Moreover, effective contacts were the most dorsal ones (contacts 2 and 3). This result may be explained by the phenotype of our patients (11.40% tremor dominant and 42.71 mixed forms) requiring current diffusion to zona incerta to relieve severe tremor (47).

In addition, STN DBS is equivalent to dopamine effect on the posture by means of rigidity relief. As expected, surgery did not ameliorate both postural instability and dysarthria. Indeed, the effect of stimulation on axial symptoms is known to be poor, their pathophysiology being different (46, 48–51). Moreover, patients can even exhibit a slight deterioration in axial symptoms, which is associated with the progression and the natural evolution of the disease (38–41, 46). An older age, intensity of axial symptoms and UDPRS II off-medication score (items 5–17) before surgery were predictive factors of dysarthria/hypophonia and postural instability after surgery (44). In our patients, the young age at surgery may explain in part the absence of such side effects. Otherwise, there was no more medication OFF FOG postoperatively confirming the benefit of DBS on dopa-sensitive symptoms.

Subsequently, we observed a considerable reduction in the daily doses of antiparkinsonian medication, up to 50% of the preoperative doses, which participated in the antidyskinetic effect of DBS. Indeed, we recorded a major reduction in dyskinesia by 66.70% and in the frequency and severity of motor fluctuations by 50%, both are known to significantly contribute to preoperative functional limitations (46) and considered by our patients as the most disabling symptoms. These results are in line with most series that reported a reduction of medication doses of 19 to 80.7%, motor fluctuation scores of 16 to 95% and dyskinesia scores of 53–92% (16, 22, 37, 42, 52–60).

There was a limited advantage of surgery on ADL assessed by UPDRS II in OFF medication state with just a trend to improvement and no effect in ON state for our patients. In several series, ADL in On medication condition had not improved or even declined at 1 year and remained stable at 5 years despite reductions in dyskinesia duration and severity, whereas in OFF medication, ADL improved by 49 to 54.2% 5 years after surgery (16, 36, 37, 42, 61, 62). However, in our series, when ADL was measured using the Schwab and England scale, we observed a significant improvement of ADL in OFF medication by 36.66%, which was in line with most series (16, 36, 37).

The good effect of STN DBS was also attested by an overall improvement of quality of life by 27% in our patients assessed by the PDQ-39 SI. This rate is in contrast with the dramatic improvement of the motor function especially the most disabling symptoms (dyskinesia and motor fluctuations) but is in agreement with previous studies. Indeed, the improvement of QOL reported varied from 30.2 to 50.6% (63, 64). Dimensions affected by DBS are subject of conflict. Some authors report that DBS ameliorates all dimensions of QOL, whereas others emphasize that the dimensions improved are those that surgery is expected to affect (ADL and mobility) but not the others (social support, cognition, and communication) (23, 64–66). Sobstyl et al. (66) demonstrated a correlation between dyskinesia and the improvement of “Mobility” and “ADL” dimensions and PDQ39 SI. This correlation can explain our results, as the significantly improved dimensions were “Mobility,” “ADL,” and “Stigma.” The improvement of “Stigma” dimension may result from the impact of dyskinesia on social life especially in our country where hyperkinetic movements are culturally not appreciated. The disappearance of dyskinesia allowed patients to be involved in social life. Over all, further studies on a large number of patients and long follow up are needed to determine the impact of DBS on QOL taking into account both motor and non-motor symptoms. The moderate improvement of QOL by STN DBS reported up to now highlights the major influence of nonmotor symptoms on quality of life (67, 68).

Various studies found no effect of STN DBS on cognitive functions while others noted worsening of verbal fluency or transient cognitive impairments (56, 69–72). In our series, we did not observe any change in cognitive and mood scales. Patients with preexisting cognitive impairment were not selected for DBS. On the other hand, all subjects were screened for depression and psychiatric disorders in order to avoid the reported potential exacerbation of mood disorders after surgery (34, 71–74).

DDS is one of the clinical aspects of Impulse Control Behaviors (ICB). It has a prevalence of 13.6% in PD and may be considered as the neuropsychiatric equivalent of levodopa-induced dyskinesia (75–77). Contrasting results of DBS on ICB are reported in the literature (78–83). Merola et al. (84) in their study of 150 consecutive PD STN-DBS-treated patients, reported only an overall trend for reduction of ICB but with significant improvement in hypersexuality, gambling and DDS after a follow up of 4.3± 2.1 years. In our series, there was no modification in the prevalence of DDS after surgery. Patients were assessed 6 to 12 months after surgery, which could be considered insufficient to appreciate the modification of their ICB. Nevertheless, new ICB may occur in some subjects with risk factors such as: younger age, female, lower dyskinesia improvement and schizoid traits of personality disorders (84). A longer follow up is needed to assess our patients for new ICB.

STN DBS can be regarded as a safe procedure in properly selected patients. Mortality and permanent morbidity are very low and surgical complications are relatively rare. However, numerous surgical, hardware-related, or infective complications may be developed after surgery or during the follow-up period, sometimes even years after the intervention for lead positioning (85). The rates of these complications are quite variable in the literature and include intracranial hemorrhage (0–10%), stroke (0–2%), infection (0–15%), lead erosion without infection (1–2.5%), lead fracture (0–15%), lead migration (0–19%), and death (0–4.4%) (13, 85–89). In our series, we did not record any cerebral hemorrhage. Major complications included infections and hardware-related ones, occurring in 8.6% of cases.

Psychiatric disorders were seen during surgery in 6 patients. This difficulty to complete the surgical procedure has been recently reported by other groups as a factor that can be time wasting and frustrating for both the patient and the surgeon, observed mainly in early series. The most frequent cause is a psychiatric disturbance of the patient, with hallucinations and impossibility to cooperate during surgery (85, 90–94).

## Conclusion

Our results showed that STN DBS is an effective treatment in Moroccan Parkinsonian patients leading to a major improvement of the most disabling symptoms (dyskinesia, motor fluctuation) and a better QOL. These findings, which are in line with those previously reported in other caucasian and asian population, showed that in carefully selected Moroccan patients with a multidisciplinary management, STN DBS is a powerful treatment that alleviates the burden of advanced PD.

## Author contributions

MR and WR participated equally to the design of the study, edited the manuscript, participated in patients' selection, peroperative microrecording, and macrostimulation testing and programming. FoB, YA, and AdM performed lead targeting and surgery. RG, NEF, and NEA: participated in surgery. MJ, MoE, MF, and NEK performed acquisition, analysis, and interpretation of radiological data. FaB and SS performed neuropsychological assessment. AbM, MA, and AS performed anesthetic monitoring. MB participated in patient's selection, per-operative neurophysiology, and macrostimulation testing. MEF participated in patients' selection and gave agreement for clinical data. SE, HeT, IEB, and HoT participated in collecting data. KE performed and edited statistical analysis. RR supervised statistical analysis. AbB participated in per-operative neurophysiology and revising the manuscript. AhB and EAB participated in critical reading of the manuscript. SA, AlB, and MY gave agreement for clinical data. AEK, AEO, FoB, and RE gave agreement for surgical data.

### Conflict of interest statement

The authors declare that the research was conducted in the absence of any commercial or financial relationships that could be construed as a potential conflict of interest. The reviewer MJ declared a past co-authorship with one of the authors AbB to the handling Editor.
